# Antenatal care strengthening for improved quality of care in Jimma, Ethiopia: an effectiveness study

**DOI:** 10.1186/s12889-015-1708-3

**Published:** 2015-04-11

**Authors:** Sarah Fredsted Villadsen, Dereje Negussie, Abebe GebreMariam, Abebech Tilahun, Henrik Friis, Vibeke Rasch

**Affiliations:** Department of Nutrition, Exercise and Sports, Faculty of Science, University of Copenhagen, Copenhagen, Denmark; Department of Public Health, Faculty of Health and Medical Sciences, University of Copenhagen, Copenhagen, Denmark; Department of Obstetrics and Gynaecology, College of Public Health and Medical Sciences, Jimma University, Jimma, Ethiopia; Department of Population and Family Health, Jimma University, Jimma, Ethiopia; JUCAN research collaboration, Jimma University, Jimma, Ethiopia; Department of Obstetrics and Gynaecology, Odense University Hospital, Odense, Denmark

**Keywords:** Antenatal care (ANC), Complex interventions, Process and effectiveness evaluation, Health system strengthening, Ethiopia, Maternal and child health

## Abstract

**Background:**

Interventions for curing most diseases and save lives of pregnant and delivering women exist, yet the power of health systems to deliver them to those in most need is not sufficient. The aims of this study were to design a participatory antenatal care (ANC) strengthening intervention and assess the implementation process and effectiveness on quality of ANC in Jimma, Ethiopia.

**Methods:**

The intervention comprised trainings, supervisions, equipment, development of health education material, and adaption of guidelines. It was implemented at public facilities and control sites were included in the evaluation. Improved content of care (physical examinations, laboratory testing, tetanus toxoid (TT)-immunization, health education, conduct of health professionals, and waiting time) were defined as proximal project outcomes and increased quality of care (better identification of health problems and increased overall user satisfaction with ANC) were distal project outcomes. The process of implementation was documented in monthly supervision reports. Household surveys, before (2008) and after (2010) intervention, were conducted amongst all women who had given birth within the previous 12 months. The effect of the intervention was assessed by comparing the change in quality of care from before to after the intervention period at intervention sites, relative to control sites, using logistic mixed effect regression.

**Results:**

The continued attention to the ANC provision during implementation stimulated increased priority of ANC among health care providers. The organizational structure of the facilities and lack of continuity in care provision turned out to be a major challenge for implementation. There was a positive effect of the intervention on health education on danger signs during pregnancy (OR: 3.9, 95% CI: 2.6;5.7), laboratory testing (OR for blood tests other than HIV 2.9, 95% CI: 1.9;4.5), health problem identification (OR 1.8, 95% CI: 1.1;3.1), and satisfaction with the service (OR: 0.4, 95% CI: 0.2;0.9). There was no effect of intervention on conduct of health professionals. The effect of intervention on various outcomes was significantly modified by maternal education.

**Conclusion:**

The quality of care can be improved in some important aspects with limited resources. Moreover, the study provides strategic perspectives on how to facilitate improved quality of ANC.

## Background

The world possess an arsenal of interventions for curing disease and save lives of pregnant and delivering women, yet the power of health systems to deliver them to those in most need is not sufficient. World Health Organization (WHO) has agitated for an urgent need to improve the performance of health systems and developed a framework for action [1]. It has been argued that efforts should be based on a continuum of care perspectives [2,3], and, thus, joint strengthening of antenatal, delivery and post-partum care is relevant.

Antenatal care (ANC) is a global health system approach to improved maternal and infant health [4-6] as ANC is considered to reduce maternal and perinatal morbidity and mortality directly through detection and treatment of illness and indirectly by improving the health behaviors of the woman. Prevention, screening and treatment for infections prevent fetal loss, preterm delivery, low birth weight and maternal and infant morbidity [5] and anti-tetanus immunization and prevention of mother-to-child-transmission of HIV (PMTCT) is known to protect infant health [7]. Moreover, iron and folate supplementation reduce anaemia, specialized treatment of severe pre-eclampsia reduce case fatality [8] and findings suggest that ANC can improve nutritional behaviors [9-12], breastfeeding practices [13,14], and use of health facility delivery [15-18].

In 2002 the WHO released new research-based guidelines for *Focused Antenatal Care (FANC),* applicable also in low-income countries [19]. The guidelines recommend four visits during pregnancy, if the pregnancy develops without complications. This four-visit model has, however not been fully implemented [20], and the quality of Antenatal Care (ANC) has in several low-income settings been assessed as low [21-24].

In Ethiopia, ANC services have been characterised by unclear guidelines and a lack of training of providers in ANC [25,26], and the health system registration practice seems poor. However, the Preventing Mother-To-Child Transmission (PMTCT) of HIV program has received high priority [27]. From 2005 to 2011, the national ANC coverage increased from 28% to 34% [28]. Urban women were more than twice as likely to attend ANC as rural women were. In urban settings, 45% attended four or more visits [29]. However, data from the study area of the present study suggest higher ANC coverage (77%), but only 7% of the women had four or more visits, and as many as 43% attended first visit in the last trimester [30].

An ANC strengthening intervention, *the Maternity Study,* was launched in the Jimma area and the surrounding urban communities in Southwest Ethiopia, to improve the quality of care and to develop strategies for improved ANC in low-income settings. The setting and prior needs assessment, using a participatory mixed method approach, has been described previously [31]. In brief, there were no official Ethiopian ANC guidelines in 2008, and the providers in Jimma did not have support to give high priority to ANC. Teaching of health professional students was given high priority, and that contributed to a lack of privacy for women. Poor user-provider interaction was a serious concern of the women which contributed to a lack of trust in the providers. The four-visit-model was only followed at the health centres, but not at the hospital. Continuity in service provision, health education, laboratory facilities, and knowledge and skills amongst staff were not adequate.

The objective of this manuscript is to describe the design of the Maternity Study and analyse the implementation process and effectiveness on quality of ANC. The intervention was designed to improve the quality of care by improving the content of care provided (proximal outcomes measured by the frequency of physical examinations, laboratory testing, tetanus toxoid (TT)-immunization, health education, conduct of health professionals, and waiting time), which then were expected to lead to better identification of health problems and increased overall user satisfaction with ANC (distal project outcomes). The study should be seen as a contribution to knowledge base of intervention research for improved quality of ANC and improved maternal and child health in low-income settings.

## Methods

### Intervention and evaluation theory

The intervention was developed using a bottom-up approach [32]. In the initial needs assessment local stakeholders, including the women, fathers, traditional birth attendants (TBAs), and health professionals were invited to express their thoughts and views about how to improve the quality of care. The needs assessment was combined with a more top-down approach [32], where a locally adapted version of the WHO guidelines for ANC [19] was used to ensure that the changes spurred in the needs assessment were aligned with international guidelines. The combined top-down bottom-up approach is in line with theories for planning of health promotion programs [33]. Engaging local stakeholders in the program planning process is crucial to align activities with local context, behaviour and culture [33,34], and previous attempts to implement maternal mortality reduction policies without involvement of local stakeholders have proven ineffective [35].

The present study constitutes a complex intervention where a range of interrelated activities are applied to address a number of current practices amongst health care providers and pregnant women. A key concept in a complex intervention approach is to study not only the effect of the intervention, but equally analyse the process of implementation, in order to understand the mechanisms of effect [36]. Further, unexpected and unintended effects are common. Therefore, to evaluate the total effect of the intervention, an exploratory approach with an extended range of outcome measures are warranted [37]. To deal with the challenge of developing, documenting and reproducing complex interventions, the British Medical Research Council (MRC) suggest a multi-staged approach to the intervention design, including several preparatory phases prior to initiating randomized controlled trials or impact evaluations [36]. In that context, the present study can be considered an exploratory trial where a special attention is directed towards feasibility, acceptability and an explorative assessment of effects.

Pawson et al. [38] criticize the idea that evaluations can give a final judgement of whether an intervention works universally and suggest that evaluations should rather study *what works for whom, in what circumstances, in what respects, and how*? And further evaluations should provide a revision of how the intervention was initially thought to work. In this process, the program theory (how the activities are expected to lead to the outcomes) becomes the unit of analysis [38].

The present study was developed with a participatory and explorative approach. We studied the degree of implementation and adaption of activities at the different sites. Moreover, the outcome evaluation included a range of outcomes to allow for measurements of effects across a range of domains. In the result section of this manuscript the implementation process and the outcome evaluation are presented separately, however in the discussion the findings are synthesised to analyse how our program theory managed or did not manage to create changes and why. We studied the effectiveness of the intervention [39], where the implementation occurred in the routine ANC services with real-world health professionals and pregnant women.

### Design of the intervention

Health facilities in Jimma and Serbo town (17 km from Jimma) were chosen as intervention sites: Jimma University Specialized Hospital (JUSH), Jimma Town Health Centre, Higher 2 Health Centre and Serbo Health Centre. Women attending ANC at Agaro Health Centre (45 km from Jimma) and *other facilities* (private clinics and clinics outside the study area) constituted the control group. According to routine registrations 2009–10, the following annual numbers of women were seen for ANC at the respective facilities: JUSH, 2144; Jimma Town, 1828; Higher 2, 1584; Serbo, 782; Agaro, 1052; and private facilities in Jimma town, 439 (these numbers include both women with urban and rural residence).

The timing of the implementation of activities and data collection are shown in Table 1. The intervention lasted for one and a half years, with the main activities implemented from July 2009 to April 2010. Thereafter, the activities were maintained through monthly supervisions until December 2010. The intervention activities are described below.

**Table 1:** **Timing of Maternity Study implementation and data collection**

	**2009**							**2010**												**2011**	
	**J**	**J**	**A**	**S**	**O**	**N**	**D**	**J**	**F**	**M**	**A**	**M**	**J**	**J**	**A**	**S**	**O**	**N**	**D**	**J**	**F**
**Implementation timeline**																					
Donation of equipment to health centres		●																			
Donation of laboratory facilities		●																			
Training of laboratory technicians		●																			
Training of health staff, seminars		●							●												
Training of health staff at antenatal care facilities			●																		
Development/revision of antenatal care guidelines		●	●	●	●	●	●		●	●											
Implementation of antenatal care guidelines					●																
Development/revision of health education material		●	●	●					●	●											
Implementation of health education material					●						●										
Seminars with traditional birth attendants					●							●			●						
Supervision of antenatal care staff			●	●	●	●	●	●	●	●	●	●	●	●	●	●	●	●	●	●	
**Data collection timeline**																					
Before-intervention survey^1^	●	●																			
After-intervention survey^2^																				●	●

^1^Women, who gave birth from April 2008 to June 2009.

^2^Women, who gave birth from January 2010 to February 2011.

Intervention health centres developed a prioritised list of needed equipment for ANC and delivery services, based on which, basic medical consumables and equipment were donated by the research project. On the list were gauze, cotton, gloves, syringes, personal protection clothing for health professionals assisting deliveries, bed sheets, blankets, oxytocine, disposable umbilical ties, blood pressure monitors, fetoscopes, stethoscopes, oxygen concentrators, suctions, and stand lamps.

Laboratory supplies and equipment were provided to make the routine tests required for ANC available at all facilities. The expected laboratory tests for three months of ANC provision were estimated, and the needed materials were given. At health centres, where the laboratory facilities were available prior to the intervention, women were charged a user fee. With the donations, we started a revolving fund to avoid implementing free services that could not be sustained. The women were asked to pay the following amounts for tests: Haemoglobin 3 Ethiopian birr (ETB) (0.17 USD), syphilis 3 ETB (0.17 USD), blood group and Rh status 4 ETB (0.23 USD), urine dip stick (including nitrite, leukocytes, blood, protein, and glucose) 3 ETB (0.17 USD). The health centres were made responsible for reimbursing these fees to new laboratory reagents. The laboratory technicians were given a two-hour refresher course at their own facility, which ended by displaying posters with the laboratory procedure for these tests.

The amount spent on equipment, consumables and laboratory supplies was 56.600 ETB (4.488 USD) at Jimma Town Health Centre, to 46.400 ETB (3.680 USD) at Higher 2 Health Centre, and 36.800 ETB (2.918 USD) at Serbo Health Centre.

All health professionals at intervention sites participated in a training package. The training aimed to improve their ANC skills but also to involve them in adapting the WHO FANC guidelines. The initial training consisted of a full-day seminar, where the outline of the new guidelines was prepared. Subsequently, the health professionals in charge of ANC received on-the-job supervision in their facilities. Six months later a subsequent two-day seminar was conducted involving all health professionals. The guidelines were revised and training was given on management of complications during pregnancy, especially local standards for referral.

Privacy guidelines were developed to set up principles for the interaction with the clients: If many health staff members or students were present during the consultation, one person should be in charge. The door should be closed, and walking in and out should be limited. The woman should be informed about the procedures undertaken, and mobile phones should be on silent mode.

A health education folder was developed based on the first training. It was written in two local languages, and it addressed the specific needs and practices of the surrounding communities with drawings from a local artist. The folder was intended as a job aid for the health professionals. It had to be handed out to the women at the first ANC visit, allowing for reflection with the partner and relatives at home. The material covered 1) an explanation of ANC service, 2) healthy behaviours during pregnancy, 3) danger signs during pregnancy, 4) birth preparedness, and 5) healthy behaviours after delivery. After the folder had been implemented and the use of it monitored, a pictogram on danger signs during pregnancy was developed, because the health professionals expressed that the folder was too detailed for the illiterate women. The pictogram was inspired by a Jamaican project [40]. Both the folder and pictogram were designed to have low printing costs: it was black ink on A4 folded paper.

The ANC guidelines included 1) a description of timing and content of the four visits 2) a list of pregnancy conditions and management procedures both for the health centres and the referral hospital level, 3) a list of the common laboratory test recommended to pregnant women, how to interpret the results and treatment regimens 4) the privacy guideline and 5) the health education material. The guidelines were distributed to all intervention facilities.

Monthly supervisions at the health centres were conducted throughout the intervention period by a research nurse employed by the research project. During the supervisions, the ANC consultations were observed and the laboratory services were monitored. All issues were written in a supervision report. The visits were unannounced, and, when possible, the health centre leader would take part in the supervision. If the health centre leader was not available, the supervision report would be discussed with the health centre leader subsequently, to facilitate local ownership and reflection on identified challenges and possible solutions.

### Program theory

In Table 2, a simplified version of the program theory of the intervention is displayed. The different activities were considered inputs that would lead to changes in the content and context of the ANC provided (proximal project outcomes), and fulfilment of these was expected to lead to both facility-related changes as well as behavioural change amongst the women (distal project outcomes). This paper is reporting the effects on the facility-related outcomes, which are better identification of health problems and increased satisfaction with care. In the long run, improvements in the distal project outcomes were expected to lead to improved maternal and infant heath and mortality; however, we did not find it realistic to assess these long term impacts with the resources of our study. The effects of the intervention on behavioural changes amongst the women will be reported elsewhere.

**Table 2:** **Program theory of the Maternity Study**

**Input**	**Proximal project outcomes**	**Distal project outcomes**	**Long-term impact**
Donation of equipment	Provision of:	Facility change:	Maternal and infant health and mortality
	- Physical examination	-Identification of health problems during pregnancy	
	- Laboratory testing		
Trainings of health staff and laboratory technicians	- TT immunization	- Satisfaction with ANC	
	- Health education		
		Behavioural change:	
ANC guidelines (incl. privacy)	Context:	- Number of ANC visits	
	- Waiting time	- Place of delivery	
Health education materials	- Conduct of health staff	- Breastfeeding	
Supervisions		- Infant preventive health care	

### Data collection

#### Implementation process

The evaluation of the implementation process at the facility level was based on monthly supervision reports.

#### Effectiveness

Questionnaire surveys were conducted at the household level, before and after the intervention amongst all women in Jimma, Serbo, and Agaro who gave birth within 12 months preceding the interview dates. Thus, the women included in the after-intervention survey were pregnant either concurrently or just after completion of the intervention (except for the supervisions). Local guides assisted to identify eligible women on a house-to-house basis in the communities. In both surveys, it was also an inclusion criterion that the women had been living in the study area for at least a year. We planned to include 300 women from the control groups and 700 from the intervention group as this would enable us to detect a difference in the proportion satisfied with the service from 50 to 60% or more with 80% power at a 5% significance level.

The questionnaire was designed to gain information on the women’s use and experience with health facilities during pregnancy, and the project outcomes were measured as reported by the women. The questionnaire was identical before and after intervention (except that place of laboratory testing was not included in the baseline survey). In order to diminish recall bias, the women were asked to remember if they had received the different services at least once during ANC. Further, the questions on blood testing were divided; 1) HIV test and 2) other blood analysis (not differentiating haemoglobin, blood group, Rh status, and syphilis tests). The questionnaire was in *Amharic* and it was pilot tested. Data were collected by trained female data collectors under daily supervision. Data were double entered by trained data entry clerks.

### Data analysis

Logistic mixed-effect regression was used to compare the change in quality of care before and after the intervention period at the intervention sites, relative to the control sites. Therefore, the regression analyses included the main effect of variable A (before- or after-intervention survey), variable B (intervention or control site), and the interaction-term between A and B. Facilities were included as random effects. Possible confounding by maternal education, parity, and marital status was assess by a comparison of crude and adjusted models. Log rank test was used to assess if the effect of the intervention was modified by maternal education. Analyses presented for 1) a full model, where all sites were divided into control and intervention sites, and 2) a submodel, in which only the intervention health centres were included (hospital excluded). Sensitivity analyses were performed to study if the effect of intervention was altered when the control group consisted only of women attending ANC at Agaro Health Centre (other facilities excluded). All analyses were performed in STATA 11.0 (StatCorp, Texas, USA).

### Ethics

Ethical permission was obtained from the Jimma University Ethical Review Committee of the College of Public Health and Medical Sciences, and permission to study and intervene on the practice at health facilities was obtained from the relevant town and zonal health bureaus as well as the hospital administration. All informants were ensured anonymity and confidentiality, and they gave informed consent after appropriate explanation of the study objectives.

## Results

### Implementation process

During the 12 months of supervision, the ANC providers seemed to gain increased interest in providing ANC: The supervisions gave continuous attention, addressed local problems, created a team spirit, and stimulated increased priority of the programme. At all sites, progress accelerated after the first two supervisions, and, without the supervisions, it is likely that the guideline and materials developed at the trainings would not have translated into practice. It was difficult to include the health centre leaders in the supervisions, partly because the supervisions needed to be unannounced, in part because ANC service was a low priority. Across the facilities, rotating ANC responsibility led to lack of continuity and sense of responsibility, and this turned out to be a major challenge for implementation.

Initially there were problems with the privacy guidelines and the health education at two health centres, however during the supervisions solutions were identified and these sustained during the intervention period. At the remaining health centre sufficient continuity was never achieved and therefore adherence to the guidelines was not consistent. The high number of health professional students was identified as the main obstacle for lack of continuity. At the hospital, the health education leaflet was relatively well introduced, but the privacy guidelines, registration procedure, and four-visit model was not successfully implemented. Supervision of the medical interns, who worked on rotation, should have been done by ANC nurses together with the managing obstetricians. However, the nurses did not have enough authority and the managing obstetricians were too busy to give priority to this work. The lack of continuity and low priority of ANC made it difficult to implement the new guidelines, and the intervention was stopped prematurely in March 2010. Subsequently, the only activity involving the health professionals at the hospital was the second training.

Throughout the intervention period, the provision of the recommended laboratory tests was a challenge. At the two health centres without laboratory facilities for ANC (but HIV tests) the equipment and the revolving funds were introduced. In March 2010, the Ethiopian Ministry of Health made it free for all women to take the ANC tests, and the revolving fund of our study ceased. Initially, the user fee was the main barrier, whereas during the free services, supply was the main challenge. Further, problems with power cuts, demotivated laboratory technicians (their workload increased without an increase in salaries), and poor communication between laboratory and ANC staff were evident.

### Effectiveness

Of 1399 women found eligible, 1357 (97%) consented to participate in the before-intervention survey. Similarly, of 2275 women found eligible for the after-intervention survey, 2262 (99%) consented to participate. In the after-intervention survey, more women were identified as eligible in Jimma town and their mean age was slightly higher, whereas no difference was found in other background variables (Table 3).

**Table 3:** **Comparison of background characteristics of the before- and after-intervention survey populations, i.e. women, who gave birth in the Jimma area within the previous 12 months** ^**1**^

	**Before intervention (N = 1357)**	**After intervention (N = 2262)**	**p-value**
Maternal age, years, mean ± SD (N)	24.5 ± 4.7 (1350)	25.1 ± 4.8 (2258)	<0.001
*Place of residence*			<0.001
Jimma	71.6 (969)	79.1 (1788)	
Serbo	6.3 (85)	6.3 (143)	
Agaro	22.2 (300)	14.6 (331)	
*Maternal education*			0.094
No school	20.0 (271)	22.8 (514)	
Primary school	46.0 (623)	43.0 (970)	
Secondary or higher school	34.1 (462)	34.3 (774)	
*Marital status*			0.228
Single	2.8 (38)	2.7 (61)	
Widow/divorced	5.1 (69)	3.9 (88)	
Cohabitating	92.1 (1247)	93.4 (2106)	
*Parity*			0.707
Para I	42.0 (563)	40.3 (903)	
Para II	26.4 (354)	26.7 (598)	
Para III	14.9 (199)	16.0 (359)	
Para IV+	16.7 (224)	17.0 (380)	
*Outcome of last pregnancy*			0.209
Singletons	97.9 (1329)	98.5 (2228)	
Twins and triplets	2.1 (28)	1.5 (34)	
*ANC attendance*			0.741
Yes	83.0 (1125)	82.1 (1856)	
No	17.0 (231)	17.9 (405)	

^1^Numbers are % (N) unless otherwise indicated and may not add up due to missing data.

Frequencies of proximal and distal project outcomes before and after the intervention at the individual sites are shown in Table 4.

**Table 4:** **Reported content of care by facility according to ANC attendants in before- (1125) and after-intervention (1856) survey** ^**1**^

	**Control sites**				**Intervention sites**							
	**Agaro HC**		**Other**		**Jimma Town HC**		**Higher 2 HC**		**Serbo HC**		**Jimma Hospital**	
	**Before**	**After**	**Before**	**After**	**Before**	**After**	**Before**	**After**	**Before**	**After**	**Before**	**After**
	**N = 250**	**N = 251**	**N = 100**	**N = 171**	**N = 215**	**N = 438**	**N = 146**	**N = 299**	**N = 55**	**N = 106**	**N = 359**	**N = 591**
*Physical examination*												
Blood pressure	96.0 (240)	94.7 (233)	92.0 (92)	93.6 (160)	97.2 (209)	96.1 (415)	97.2 (140)	96.6 (288)	94.6 (52)	94.3 (99)	98.0 (350)	96.4 (570)
Weight measurement	96.0 (240)	94.4 (243)	96.0 (96)	96.4 (162)	98.1(211)	97.7 (426)	99.3 (144)	99.3 (296)	94.6 (52)	97.2 (103)	98.3 (353)	98.3 (578)
Abdominal examination	66.8 (167)	89.9 (223)	83.0 (83)	94.1 (160)	86.5 (185)	97.7 (424)	86.3 (126)	93.7 (280)	96.4 (53)	99.1 (105)	95.8 (344)	97.3 (573)
*Laboratory tests*												
HIV test	92.8 (232)	95.9 (233)	82.0 (82)	92.4 (157)	95.8 (204)	97.5 (425)	95.2 (139)	98.3 (294)	76.4 (42)	98.1 (104)	96.9 (348)	98.1 (576)
Blood test, other	41.5 (103)	41.6 (87)	65.7 (65)	82.6 (138)	79.3 (168)	88.4 (381)	69.7 (101)	89.5 (265)	7.4 (4)	78.1 (82)	80.6 (286)	87.7 (511)
Urine analysis	42.8 (107)	72.7 (178)	69.7 (69)	90.6 (155)	81.9 (176)	97.2 (423)	71.9 (105)	96.0 (287)	34.6 (19)	67.6 (71)	93.6 (336)	96.8 (571)
*TT immunization*	95.2 (238)	84.6 (208)	91.0 (91)	88.9 (157)	97.2 (209)	94.2 (408)	95.2 (138)	94.6 (282)	76.4 (42)	98.1 (104)	85.7 (306)	90.9 (531)
*Health education topics*												
Danger signs during pregnancy	30.8 (77)	37.1 (89)	46.5 (46)	50.6 (85)	38.9 (81)	70.0 (298)	29.9 (43)	66.9 (196)	18.5 (10)	86.6 (90)	44.1 (156)	56.9 (327)
Need for health facility delivery	38.4 (96)	55.3 (136)	58.0 (58)	73.1 (125)	54.0 (115)	82.6 (356)	65.1 (95)	83.4 (241)	30.9 (17)	90.6 (96)	62.1 (223)	68.7 (399)
Nutritional needs during pregnancy	39.8 (99)	56.9 (140)	66.0 (66)	68.4 (117)	59.4 (127)	82.1 (357)	71.2 (104)	81.5 (242)	47.3 (26)	87.6 (96)	64.1 (230)	67.2 (394)
Breastfeeding	46.8 (117)	61.4 (151)	59.0 (59)	71.2 (121)	58.2 (124)	83.8 (363)	58.2 (85)	83.6 (244)	47.3 (26)	90.6 (96)	59.6 (214)	66.8 (392)
HIV/AIDS	64.0 (151)	55.2 (133)	51.1 (48)	67.3 (101)	54.0 (114)	77.8 (291)	50.7 (74)	84.3 (215)	35.3 (12)	86.5 (77)	61.1 (206)	71.6 (384)
*Health staff practices*												
Discomfort caused by students	26.0 (64)	19.7 (49)	13.3 (13)	5.9 (10)	23.0 (49)	15.2 (66)	15.2 (22)	12.8 (38)	25.5 (14)	19.8 (21)	36.8 (131)	21.0 (123)
Poor conduct of health staff	5.3 (13)	3.3 (8)	4.0 (4)	1.2 (2)	3.7 (8)	1.4 (6)	0	2.0 (6)	18.2 (10)	0.9 (1)	6.7 (24)	2.0 (12)
*ANC surroundings*												
Waiting less than 1 hour pre service	83.7 (204)	72.5 (182)	89.0 (89)	87.7 (149)	83.3 (175)	88.1 (384)	72.6 (106)	82.9 (247)	98.2 (54)	95.3 (101)	56.1 (198)	67.0 (394)
*Distal project outcomes*												
Health problem identified	13.3 (33)	9.6 (24)	15.6 (15)	20.5 (35)	12.6 (26)	20.0 (87)	12.6 (18)	21.5 (64)	11.1 (6)	18.3 (19)	14.9 (52)	19.3 (113)
Not satisfied with service	17.2 (42)	14.4 (35)	6.1 (6)	1.2 (2)	6.1 (13)	2.6 (11)	2.2 (3)	2.4 (7)	25.9 (14)	1.9 (2)	9.9 (35)	4.8 (27)

^1^Numbers are % (N) and may not add up due to missing data.

At control sites, there was increased coverage of all health education topics, but, at the intervention health centres, the increase was significantly larger (Table 5). For health education on danger signs, the coverage increased from 35% to 43% at control sites, whereas, at intervention health centres, the increments were from 38 to 65%, resulting in an OR of 3.9 (95% CI: 2.6;5.7). At control sites, there was a decrease in frequency of TT immunization, and this decrease was not present at intervention sites. There was a significantly larger increase in both blood tests other than HIV and urine tests at intervention sites compared to control sites (OR 2.9, 95% CI: 1.9;4.5) and (OR 1.8, 95% CI: 1.1;3.0) respectively. The intervention increased in the proportion of women waiting less than one hour (OR 2.6, 95% CI: 1.6; 4.3). There was no effect of the intervention on the frequency of physical examination performed, conduct of health professionals, and discomfort caused by students. There was effect of intervention on health problem identification, and thus more women at intervention health centres reported health problems after intervention (OR 1.8, 95% CI: 1.1;3.1). Overall, only 14% of the women at control sites and 9% at intervention sites were not satisfied with the ANC service before the intervention. The proportion of women who were not satisfied with ANC service decreased more at the intervention sites than it did at the control sites (OR 0.4, 95% CI: 0.2;0.9). Adjustment for maternal education, parity, and marital status did not change the results.

**Table 5:** **Effect of intervention on reported content of care** ^**1**^

	**% Control**		**% Intervention (All sites)**		**% Intervention (HC only)**		**Odds ratio** ^**2**^	
							**(All sites)**	**(HC only)**
	**Before**	**After**	**Before**	**After**	**Before**	**After**		
	**N = 350**	**N = 422**	**N = 775**	**N = 1434**	**N = 416**	**N = 843**		
*Physical examination*								
Blood pressure	94.7	94.2	97.4	96.4	96.9	96.1	0.8 (0.4;1.8)	0.9 (0.4;2.2)
Weight measurement	96.0	95.2	98.2	98.3	98.1	98.2	1.3 (0.5;3.3)	1.3 (0.4;4.0)
Abdominal examination	71.4	91.6	91.5	96.8	87.7	96.4	0.7 (0.4;1.3)	0.9 (0.5;1.7)
*Laboratory tests*								
HIV test	89.7	94.4	94.8	98.0	93.0	97.9	1.2 (0.6;2.6)	1.6 (0.7;3.6)
Blood test, other	48.4	59.9	73.0	87.6	66.4	87.5	2.1 (1.4;3.1)	2.9 (1.9;4.5)
Urine analysis	50.4	80.1	86.1	94.6	72.1	93.1	1.3 (0.8;2.1)	1.8 (1.1;3.0)
*TT immunization*	94.0	86.3	90.0	93.2	93.7	94.9	3.7 (2.0;6.9)	3.1 (1.5;6.3)
*Health education topics*								
Danger signs during pregnancy	35.2	42.7	38.2	65.2	33.0	71.0	2.3 (1.6;3.3)	3.9 (2.6; 5.7)
Need for health facility delivery	44.0	62.6	58.2	77.6	54.8	83.9	1.2 (0.9;1.8)	2.2 (1.5;3.2)
Nutritional needs during pregnancy	47.3	61.6	62.9	76.3	61.9	82.6	1.1 (0.8;1.6)	1.7 (1.2;2.6)
Breastfeeding	50.3	65.4	58.1	77.2	56.8	84.6	1.3 (0.9;1.9)	2.3 (1.5;3.4)
*Health staff practices*								
Discomfort caused by students	22.4	14.1	28.1	17.4	20.6	14.9	0.9 (0.6;1.4)	1.1 (0.7;1.8)
Poor conduct of health staff	4.9	2.4	5.4	1.8	4.3	1.6	0.6 (0.2;1.6)	0.7 (0.2;2.1)
*ANC surroundings*								
Waiting less than 1 hour pre service	84.9	78.6	69.8	78.9	81.5	87.1	2.6 (1.7;4.1)	2.6 (1.6;4.3)
*Distal project outcomes*								
Health problem identified	14.0	14.1	13.6	19.9	12.4	20.3	1.6 (1.0;2.5)	1.8 (1.1;3.1)
Not satisfied with service	14.0	9.1	8.6	3.4	7.5	2.3	0.5 (0.3;1.0)	0.4 (0.2;0.9)

^1^Numbers are % before and after the intervention period at control or intervention sites, and OR with 95% CI.

^2^The odds ratios (95% CI) express the change from before to after at the intervention sites relative to the change at the control site.

The effects of intervention on HIV test, other blood tests, TT-immunization, waiting, and health education topics were significantly modified by maternal education (Table 6). The effects of the intervention on blood tests other than the HIV test decreased with increasing education, as the OR was 2.9, 2.2, and 2.1 for women with no, primary, and secondary schooling, respectively (p for interaction 0.009). The effect of intervention on health education on the need for health facility delivery, nutritional needs, and breastfeeding seemed to be larger amongst women with primary and secondary education than it was amongst women with no education. Whereas, the effect of health education on danger signs during pregnancy was lowest amongst women with secondary education. Waiting more than one hour prior to service was reduced amongst women with primary or higher educational level, whereas no effect was seen in waiting for women with no education.

**Table 6:** **Effects of intervention expressed as odds ratios (95% confidence interval) stratified by maternal education**

	**No school**	**Primary school**	**Secondary or higher school**	**p-value of interaction**
HIV testing	2.3 (0.6;9.5)	1.5 (0.4;6.0)	2.3 (0.3;16.2)	p < 0.001
Blood test, other	2.9 (1.1;7.7)	2.2 (1.2;4.1)	2.1 (1.0; 4.4)	p = 0.009
TT immunization	5.3 (0.9;32.1)	4.1 (1.4;12.2)	1.8 (0.4;7.0)	p = 0.022
Waiting less than 1 hour pre service	1.9 (0.5;6.4)	2.4 (1.2; 4.9)	4.4 (1.7;11.2)	p = 0.012
Health education topics				
Danger signs during pregnancy	3.4 (1.2;9.6)	5.2 (2.9;9.4)	2.8 (1.5;5.4)	p = 0.003
Need for health facility delivery	1.0 (0.4;2.4)	3.3 (1.8;6.1)	2.0 (1.0;3.9)	p < 0.001
Nutritional needs during pregnancy	0.5 (0.2;1.4)	1.9 (1.0;3.4)	2.7 (1.4;5.2)	p = 0.016
Breastfeeding	1.1 (0.4;2.6)	2.3 (1.5;5.2)	3.1 (1.6;6.3)	p = 0.002

In the after-intervention survey, the ANC attendants reported that 56% at both Jimma Town and Higher 2 Health Centre, 69% at Serbo Health Centre, and 33% at the hospital received the health education folder/pictogram. Of those who received the material 97% assessed the material to be good, whereas 3% found it average (data not shown). When the folder/pictogram was given to the women the likelihood that health education would be provided during service was more than double than if no folder/pictogram was given (Table 7). Finally, the women who were given a folder/pictogram to take home had better understanding of the health education provided during service than the women who did not get the material had (OR: 2.1 95% CI: 1.4;3.0).

**Table 7:** **Health education provided during service and understanding of the health education among ANC attendants at intervention sites in the after-intervention survey (n = 1434) by folder/pictogram**

***Health education provided during ANC***	**No folder/pictogram** ^**1**^ **(N = 679)**	**Folder/pictogram** ^**1**^ **(N = 617)**	**OR 95% CI crude**	**OR 95% CI adjusted** ^**2**^
Danger signs during pregnancy	54.4 (358)	77.7 (475)	2.6 (2.0;3.4)	2.6 (2.0;3.3)
Need for health facility delivery	70.1 (463)	86.9 (532)	2.4 (1.8;3.3)	2.3 (1.7;3.2)
Nutritional needs during pregnancy	69.3 (467)	86.0 (528)	2.4 (1.8;3.2)	2.3 (1.7;3.1)
Breastfeeding	69.3 (463)	86.7 (533)	2.4 (1.8;3.2)	2.3 (1.7;3.2)
*Understanding the health education provided during ANC*	82.3 (518)	91.6 (546)	2.2 (1.5;3.1)	2.1 (1.4;3.0)

^1^Numbers are % (N) and may not add up due to missing data.

^2^Adjusted for: Parity, maternal education, and marital status.

In the sensitivity analysis, where the effects of intervention were studied with only Agaro Health Centre as the control site, the results were similar. The directions of the effects were all the same, but the effects were stronger at intervention health centers for other blood tests (OR: 3.8, 95%. CI: 2.4; 6.2), TT immunization (OR: 4.5 (1.9;10.4), and problems identified (OR: 2.6, 95%. CI: 1.3; 5.0).

## Discussion

In the result section we have analysed the implementation process and the outcomes of a complex, health system strengthening intervention for improved ANC in a low-income setup. In the following we will synthesise the main findings and illuminate the mechanisms of the intervention to study what worked where, under which circumstances and contextualize the findings with previous insight in the field.

### Proximal project outcomes

The intervention was successful in increasing the health education provision during ANC, maybe because the material was developed together with the health professionals to address needs and solutions that were applicable in the specific context. The material served as a job aid, and thus the staff used it to remember and prioritise topics. The effects of the intervention on coverage of health education topics were modified by educational level and reflected whether the women received the pictogram about danger signs (most likely women with no education) or the folder covering more topics (most likely women with education). Thus, the differential pedagogical means to health education according to the needs of the woman seemed useful in this setup. The after-intervention survey results revealed that the attitudes towards the folder/pictogram were positive and that the introduction enhanced the understanding of the health education. Health education and counselling are essential components of the international guidelines for ANC, but time allocated to these activities is often short [15,41,42], and less than 50% of women participating in ANC in an Ethiopian study received counselling on birth preparedness [43]. Intervention research from various settings has shown that it is possible to scale up the health education provided during ANC service by training the health professionals and developing job aids in the form of written material or pictograms [40-42]. Thus, it is plausible that the initial low levels of health education and the success in translating intervention activities into improved health worker practices are not specific to Jimma. For the future, it will be interesting to assess if the increased health education was translated into changed health and care-seeking behaviours.

Good health education is also related to the user–provider interaction. It has been shown elsewhere that health care provision based on empowerment and empathy is associated with good health outcomes [44,45]. A recent study from Ethiopia found that lack of privacy increased the likelihood that women would report poor provider empathy, and that empathy in general was low in primary health care centres [46]. The privacy guidelines of the present study aimed to improve the interaction, but it turned out difficult. This might explain why there was no effect on the proximal project outcomes regarding conduct of health professionals and discomfort caused by students. A reason for the lack of improvements in this area could be that the privacy guidelines were focused on principles for privacy, but not clear about how to negotiate consensus between health care providers if for example more than one health care provider was present. Further, the weak engagement of the local leaders (for example the lack of participation of health centre leaders in supervisions) might be a key issue. It could be argued that change of user-provider interaction in this clinical setting involves a redistribution of costs and benefits between the involved parties and thorough analysis of power, interests and values would be important to address [47].

The intervention had positive effects on laboratory tests other than the HIV test. The free ANC laboratory services introduced by the Ethiopian Ministry of Health during the study could have led to the increased testing at both intervention and control sites. After the intervention, more women from the control site in Agaro went to private facilities for testing (30%) than women from intervention sites (10%) did, indicating better performance by the intervention facilities. However, there was no information on these proportions in the before-intervention survey, and thus it is not possible to study changes in laboratory coverage at the public facilities. Previous studies on quality of ANC services in Sub-Saharan Africa have also documented nonexistent or poor coverage and low-quality laboratory facilities for medical conditions other than HIV [21,23]. The effect of the intervention should be understood in light of the donations provided, and could thus be considered a direct reflection of our own inputs. However, the laboratory services were kick started with three months input for a revolving fund, and the funding was sustained after the donation expired. Having the equipment available is not always enough for actions to happen and the participatory approach including trainings and regular supervisions might have had significant implication.

### Distal project outcomes

The intervention had a positive effect on health problem identification during ANC service, which is a precondition for providing treatment for pregnant women suffering illness and infectious diseases, which is known to improve also infant health and survival [5]. The increased identification of problems might reflect improved diagnostic skills of health professionals. We consider this change a result of the trainings, implementation of the guidelines, and the increase in laboratory tests. A Jamaican intervention study aiming to reduce eclampsia cases by improved identification, referral, and management of preeclampsia found that more women with preeclampsia were referred to specialised facilities, whereas the eclampsia rate declined [48]. Thus, also in the eclampsia study, the proportion of women with problems increased with improved organisation, and this resulted in improved maternal health. In the FANC guidelines, it is expected that around 25% of women will be in need of specialised care [19]. Therefore, the increase in health problem identification at intervention sites from 14% before to 20% after the intervention seems to be an appropriate, though possibly not sufficient, increase.

The measurement of satisfaction with care was relevant as an overall assessment of the women’s evaluation of the care received, and it was expected to improve when the content of care improved. However, satisfaction has previously been shown to be dependent on the expectations towards care [49,50]. In the present study, a relatively small proportion of women reported being not satisfied with ANC before the intervention, and it is also previously recognised that respondents tend to report favourable to questions of perceived quality of care or satisfaction [51]. Therefore, such measures should be interpreted with care, and they are best used in relative rather than absolute terms [51]. The effect of intervention was measured in relative terms, and it showed a small positive effect on satisfaction.

### Study strengths and limitations

At the national level in Ethiopia, the ANC services, including coverage, number of visits, and content of care, seemed to have improved from 2005 to 2011 [29,52]. Thus, the improvements studied at the intervention sites were occurring in a system, where general changes for the better occurred concurrently. This highlights the importance of the control group, which allowed us to determine which changes were brought about by the intervention and which would have occurred anyway. Agaro Health Centre was chosen as the control site because it shared some of the urban assets with the intervention sites, but also because it was located an hour’s drive away from Jimma. Moreover, women attending other facilities were also included as control site, as these facilities they were less likely to have information about the study. In the sensitivity analysis where other facilities were not included in the control group, the benefits associated with the intervention gained strength, but overall conclusions were not changed. Adjustment for background variables did not alter the results and thus the differences in measured background variables did not seem to confound the assessment of the effects of the intervention.

The changes in quality of care in this study were measured as reported by the women, and were thus measures of perceived content and quality of care. As mentioned previously, perceived quality of care and satisfaction should be interpreted with care [51]. However, there seems to be an increasing acceptance of user evaluations of care and its importance for health care utilisation [20,49]. In particular, for reproductive health care, user perspectives are very interesting for understanding how high ANC coverage could be translated into improved coverage of health facility delivery [15-17,20,53,54]. The analysis of effect of intervention on identification and management of maternal health problems could have been strengthened if we had routine data from the facilities. The routine registration was very inconsistent, and it was not monitored and didn’t seem to be subject to much reflection. We planned to include routine data from the ANC charts kept at the facilities in present study, and the data were collected as consistently as possible before, midway, and after the intervention. We tried to identify the charts prospectively and retrospectively, but they were misplaced, lost, or reused. Thus, the validity was too poor for the data to be included in the present study.

It is obvious that when a woman reports that blood pressure was measured, we still have no information about the blood pressure value or the actions taken, if abnormal. Nevertheless, the different contents of care are important precursors for correct treatment, and the changes in health problem identification are therefore considered important.

The evaluation only showed positive effects of intervention on some, but not all outcomes, and this specificity of effects could be understood by the process of implementation. For example, the intervention was not fully implemented at the hospital, and when this site was included in the analysis, only few outcomes were significant. In contrast, at the health centres, the intervention was better implemented, and the analyses with only these show stronger effects.

To conduct a survey of this size is a logistical challenge in a setup, with no universal registration of deliveries and poor registration of addresses. Prior to data collection, it was not possible to get a reliable estimation of how many pregnant women there were in these urban areas. The before- and after-survey data were collected according to similar protocols, but the experiences from the before-survey had possibly improved the skills of both the research team and the data collectors. Except for the difference in number included, especially from Jimma, there was no important difference in background characteristics in the before- and after-intervention survey.

The real-life setting of this study makes it likely that the findings could also have relevance in other contexts, however, not without adaption and user involvement and ownership.

## Conclusions

This article reported on the design, implementation, and effectiveness of a health system strengthening intervention for improved ANC in a low-income setup. The intervention was successful in improving both the coverage of laboratory tests and health education. The more distal outcomes of health problem identification and overall satisfaction were also improved. However, it was not possible to show any improvements in the user–provider interaction (measured by discomfort due to students and poor conduct of health professionals) and here more comprehensive changes in priorities are needed. If these components could be improved, we believe the effects of the intervention on the distal outcomes could have been stronger.

The health system in Jimma is dealing with competing needs and priorities with very limited resources. Provision of ANC service has not been given high priority, and the inadequate quality of ANC put women’s trust in the health system at risk [31]. With this intervention study, we have shown that quality of ANC can be improved in some important aspects with few resources. Many of the needs identified in this study seems relevant for other low-income settings, and the experiences from this study present strategic perspectives on how to facilitate the improved quality of ANC with limited resources. Based on this innovative insight, it could be interesting to continue with a larger scale implementation, allowing for evaluation of the long term impact on maternal and child health in a stronger cluster randomised design.
